# Growth Hormone Deficiency in a Patient with Becker Muscular Dystrophy: A Pediatric Case Report

**DOI:** 10.1155/2013/684249

**Published:** 2013-01-14

**Authors:** Valeria Calcaterra, Annachiara Malvezzi, Rossana Toglia, Angela Berardinelli, Elena Bozzola, Mauro Bozzola, Daniela Larizza

**Affiliations:** ^1^Department of Internal Medicine, University of Pavia, 27100 Pavia, Italy; ^2^Department of Pediatrics, IRCCS Policlinico S. Matteo Foundation, 27100 Pavia, Italy; ^3^IRCCS C. Mondino Foundation and University of Pavia, 27100 Pavia, Italy; ^4^Pediatric and Infectious Disease Unit, Department of Pediatrics, Bambino Gesù Children's Hospital, 00165 Rome, Italy

## Abstract

*Objective*. To describe a biochemical growth hormone (GH) deficiency and to evaluate therapeutic result in a six-year-old male with Becker muscular dystrophy (BMD). *Methods*. GH peak was evaluated after response to arginine and insulin. Bone age was evaluated according to Greulich and Pyle method. *Results*. The GH-supplementary therapy was very effective in terms of growth gain. *Conclusion*. The possibility of a growth hormone deficiency and treatment with GH in patients with BMD cannot be excluded, especially considering the good therapeutic response.

## 1. Introduction

Becker muscular dystrophy (BMD) is a condition due to mutations in the gene that expresses the dystrophin protein, located in X chromosome. Dystrophin is present in the muscle cytoskeleton and its failure causes sarcolemma instability and disruption. This seems to be the main factor of myopathy occurring in DMB, resulting in progressive muscle weakness, especially of the upper districts and dilated cardiomyopathy [1, 2].

A gradual slowing down of growth is described in muscular dystrophies, particularly in Duchenne's; however, this does not seem related to growth hormone deficiency [3–6].

We report a case of BMD with GH-deficient short stature who showed a good growth response to recombinant human GH therapy.

## 2. Case Presentation

D. R. was born at 40 weeks' gestation with a birth weight of 3000 g. At 18 months of age, Becker muscular dystrophy was diagnosed (deletion of exons 45–47 in the locus of dystrophin).

At the age of six yr the patient was referred to our Endocrinology Unit for short stature and delayed bone age in relation to chronological age (bone age according to Greulich and Pyle method: 42 months).

At first evaluation D. R. showed the following: height 99.4 cm (−3.1 SDS) with height genetic target of 165.7 cm (10th centile); weight 17.6 kg (3th–10th centile); BMI 17.9 kg/m^2^ (75th–90th centile); pubertal stage 1 according to Tanner [7, 8].

Initial laboratory studies revealed the following: IGF 53 ng/mL (25th percentile for age and gender), normal thyroid function with absence of autoantibodies, negativity of anti-endomisium and anti-tissue tranglutaminase, normal adrenal function. The evaluation of GH secretion showed a partial GH deficiency (GH peak response to arginine: 4.0 ng/mL; GH peak response to insulin: 8.7 ng/mL). After oral glucose test a normal tolerance was found. Magnetic resonance imaging of the hypothalamic-pituitary region was normal.

At the age of six yr and four months, GH replacement therapy was started at the dose of 0.027 mg/kg/day. The GH-supplementary therapy was very effective in terms of growth gain (Table 1).

At the last evaluation, at the age of 11, the patient showed the following: height 131.4 cm (5th centile; 1.9 SDS); height velocity 1.8 SDS; weight 29.9 kg (25th centile), BMI 17.4 kg/m^2^ (25th–50th centile), pubertal stage 2 according to Tanner.

During followup the child maintained stability of the clinical frame (characterized by mild weakness, fatigue, myalgias, and hypertrophy of the calves), neuro- and cardio-respiratory functions, and metabolic parameters.

## 3. Conclusion

Becker muscular dystrophy is a neuromuscular recessive disease related to X chromosome caused by qualitative and quantitative alterations of the dystrophin. BMD affects about 5/100,000 newborns; however, the high premature mortality explains why the disease has such a low incidence (2.4/100,000) [1, 2]. From a clinical point of view, affected patients present a progressive loss of strength, which is more marked in charge of proximal districts, particularly in the proximal pelvic girdle, and dilated cardiomyopathy, which often plays a decisive role in the evolution of the disease. Clinical evolution shows sural triceps pseudohypertrophy; femoral quadriceps can occur to be both hypo- and hypertrophied. Compared to the Duchenne muscular dystrophy, the BMD hardly presents respiratory compromisation. 

In muscular dystrophies, especially in DMD, a peculiar pattern of growth has been described, which is characterized by regular weight and length at birth and a slow progressive statural slowdown in the first years of life [3, 4]. Short stature does not seem to be related to GH deficiency or GH-IGF axis alterations [5]; instead, it might be related to several other factors such as reduced physical activity [4], abnormal response to GH [5], SHOX (short stature homeobox-containing) gene alterations, which is mapped to chromosome Xq22, therefore potentially involved in the anomalies of the nearly region Xq21, which contains the coding gene for the dystrophin [3, 4, 6].

In our report, short stature is related to GH deficiency. GH deficiency may be total or partial, isolated, or associated with other abnormalities of pituitary hormones. In childhood the disease is usually idiopathic but may be secondary to morphological abnormalities, malformations, or brain tumors. Our patient presents a partial GH secretion deficiency, which is not associated with other secretory anomalies and is not secondary to alterations of the hypothalamic-pituitary region. The growth hormone therapy had a beneficial effect in terms of growth gain, with stability of the neuromuscular disease, but we cannot define any specific positive effects on muscle. In the literature, the association between BMD and GH-deficiency has not been previously reported; other studies will help to determine if this association is casual or if in BMD growth hormone secretion and response to treatment are different from Duchenne Muscular Dystrophy. 

In conclusion, the possibility of growth hormone deficiency and treatment with GH in patients with BMD cannot be excluded, also considering the beneficial effect of the therapy.

## Figures and Tables

**Table 1:** Clinical, hormonal, and radiological parameters of the patient during GH therapy.

Parameters	Time of therapy				
	Start	1 year	2 years	3 years	4 years
Age	6 yr and 4 m	7 yr and 7 m	8 yrand 6 m	9 yr and 5 m	10 yr and 5 m
Height					
(cm)	100	108.5	115	122	128.5
(SDS)	−3.66	−3.01	−2.72	−2.34	−2.07
Weight (Kg)	17.5	20.4	22.4	25.8	29.3
BMI					
(kg/m^2^)	17.9	17.3	16.9	17.3	17.7
(SDS)	0.62	0.38	−0.03	−0.11	−0.21
Height velocity					
(cm/yr)	Unknown	6.65	7.52	7.22	6.59
(SDS)		1.53	2.56	2.53	2.12
Bone age	42 months	4–6 yr	6 yr	8-9 yr	11 yr
IGF 1					
(ng/dL)	53	68.4	174.0	267.0	221
(centile*)	25th	25th–50th	50th–95th	50th–95th	50th–95th

*Centile for age and sex [7].
